# The hemagglutinin-neuramidinase protein of Newcastle disease virus upregulates expression of the TRAIL gene in murine natural killer cells through the activation of Syk and NF-κB

**DOI:** 10.1371/journal.pone.0178746

**Published:** 2017-06-14

**Authors:** Ying Liang, De-Zhi Song, Shuang Liang, Zeng-Feng Zhang, Ling-Xi Gao, Xiao-Hui Fan

**Affiliations:** 1 Department of Microbiology, School of Preclinical Medicine, Guangxi Medical University, Nanning, Guangxi, China; 2 Department of Pharmaceutical and Medical Equipment, Trading Center of Guangxi Public Resources, Nanning, Guangxi, China; Ohio State University, UNITED STATES

## Abstract

Newcastle disease virus (NDV) is responsible for tumoricidal activity *in vitro* and *in vivo*. However, the mechanisms that lead to this activity are unclear. Natural killer cells are able to induce apoptosis of tumor cells through multiple pathways, including the tumor necrosis factor-related apoptosis-inducing ligand-death receptor pathway. We previously showed that exposure of NK and T cells to NDV resulted in enhanced tumoricidal activity that was mediated by upregulated expression of the TRAIL gene, *via* an interferon gamma -dependent pathway. Other pathways involved in the upregulated expression of TRAIL are yet to be identified. In the current study, we used mice in which the IFN-γ receptor one gene was inactivated functionally. We identified an IFN-γ-independent TRAIL pathway in the NDV-stimulated NK cells. Hemagglutinin-neuramidinase induced expression of the TRAIL gene in IFN-R1^-/-^ NK cells by binding to the NKp46 receptor. This upregulation was inhibited by pretreatment of NDV with a neutralizing monoclonal antibody against HN, or desialylation of NK cells. Phosphorylation of spleen tryosine kinases and IκBα was increased in HN-induced IFN-R1^-/-^ NK cells. Treatment with the HN neutralizing monoclonal antibody, pharmacological disialylation, or a Syk inhibitor decreased Syk and IκBα phosphorylation levels. We concluded that killer activation receptors pathway is involved in the IFN-γ-independent TRAIL expression of NDV-stimulated NK cells, and these are activated by Syk and NF-κB.

## Introduction

The innate immune response, known as one of key mechanisms of host defense, can eliminate tumor cells during the early stages of tumor formation, and is required for the maintenance and renewal of tumor-specific T cell immunity. These processes involve the activation and proliferation of immunocytes, and the secretion of functional cytokines. Viral therapy, using oncolytic viruses, can effectively improve a patient’s innate immunity against tumor cells [1–4]. The avian paramyxovirus Newcastle disease virus (NDV) possesses antitumor activity and is virtually safe for the host [5, 6], as demonstrated in animal models and in several clinical trials [7–12]. Numerous investigations have been conducted into the antitumor mechanisms of NDV, including its selective replication in tumor cells and its ability to stimulate immunoreactive cells [2, 13–15]. NDV stimulates innate antitumor immune responses by enhancing the cytotoxic functions and cytokine production of dendritic cells (DCs), natural killer (NK) cells, and macrophages [16–19]. The cytotoxicity of NK cells is dependent upon the release of perforin and granzymes, the secretion of tumor necrosis factor alpha (TNF-α) and the expression of apoptosis-inducing ligands such as Fas ligand (FasL) and TNF-related apoptosis-inducing ligand (TRAIL) which result in the apoptosis of tumor cells *via* ligand-receptor reactions. Unlike other apoptosis-inducing ligands, TRAIL is safe to the host as its receptors, TRAIL-R1 and TRAIL-R2, are only expressed on tumor cells. It was previously shown that NDV upregulates TRAIL expression in immunoreactive cells [17, 20, 21]. NDV induces cytotoxicity in human monocytes through the production of TRAIL [22]. The enhanced antitumor cytotoxicity of mouse macrophages and human peripheral blood mononuclear cells (PBMC), respectively, following exposure to NDV correlates with the production of increased amounts of cytokine production including interferon-γ (IFN-γ), interleukin-2 (IL-2), interleukin-12 (IL-12) and tumor necrosis factor-α (TNF-α) [23, 24]. We previously showed that exposure of NK cells to NDV results in the upregulation of TRAIL in these cells, and enhanced cytotoxic funtions against tumor cells [18]. However, the mechanisms by which immunocytes sense stimuli and trigger intracelluar signal pathways involved in NDV-induced TRAIL upregulation are not well understood. We observed increased IFN-γ levels when NDV was mixed with NK cells, which in turn were able to sense IFN-γ and somehow upregulate expression of the TRAIL gene. However, the IFN-γ pathway that mediated this upregulation was not completely inhibited by neutralizing IFN-γ, suggesting that the existence of a IFN-γ-independent signaling mechanism. Jarahian et al found that direct activattion of NK cells by Hemagglutinin-neuraminidase (HN), the viral envelope protein of NDV,contributes to the antitumor effects of NDV and HN serves as a ligand structure for the natural cytotoxicity receptors NKp44 and NKp46, which are killer activation receptors (KARs) in human NK cells [25].We hypothesized that these interactions between HN and KARs may be as one of IFN-γ-independent signaling mechanisms that mediated the upregulation of TRAIL in NDV-induced NK cells. To investigate this, we used mice lacking the IFN-γ receptor one gene (IFN R1^-/-^), as the cells in these mice were insensitive to the antiviral activity of IFN-γ [26]. We assessed whether TRAIL was expressed in NDV-induced splenic NK cells of IFN R1^-/-^ mice, and whether interactions between HN and NKp46 affected TRAIL expression. We also investigated which intracelluar signal transduction factors were involved in the upregulation or TRAIL expression. We attempted to show that KARs play an important role during the early stages of the innate immune response against tumor cells.

## Material and methods

### Mice, virus, and reagents

We purchased B6.129S7 IFN-R^-/-^ mice and control mice from Nanjing Biomedical Research Institute (Nanjing, China). All animal protocols were approved by the Institutional Animal Care and Use Committee of Guangxi Medical University. The avirulent, non-lytic NDV strain 7793 (NDV 7793) was obtained from our laboratory. A stock of infectious virus was propagated in embryonated chicken eggs. The allantoic fluid was collected from eggs and centrifuged (300–400×*g*, 30min, 4°C), and then subjected to ultracentrifugation (50,000×*g*, 60min, 4°C). The pellet was resuspended in phosphate-buffered saline (PBS) and purified twice using a 35% sucrose gradient and ultracentrifugation (97,000×*g*, 60min, 4°C). Purified virus was resuspended in PBS containing 0.1% EDTA. NDV titers were determined using hemagglutination tests, with a single hemagglutination unit (HU) defined as the lowest virus concentration leading to visible agglutination of chicken erythrocytes.

The pET-HNa vector, encoding the HN gene of NDV, was used to express recombinant HN in *Escherichia coli* strain BL21 (DE3), which was then purified. The pET-F vector, encoding the F gene, was used to express recombinant F in *Escherichia coli* strain BL21 (DE3), which was then purified. EZ-Sep Lymphocyte Separation Medium for mouse was purchased from DaKewe (Shengzheng, China).A magnetic-activated cell sorting (MACS) separator, a mouse NK cell isolation kit, a monoclonal antibody (mAb) against CD49 conjugated to FITC, and rat IgM conjugated to FITC were purchased from Miltenyi Biotec (Germany). Recombinant mouse IFN-γ was purchased from PeproTech (Rocky Hill, NJ, USA). A neutralizing mAb against NDV HN, a mAb against NKp46 conjugated to PE, a mAb against TRAIL conjugated to PE, rat IgG 2a conjugated to PE, a mAb against spleen tyrosine kinases (Syk), a mAb against IκBα and a mAb against phosphorylated IκBα (pIκBα) were purchased from Santa Cruz Biotechnology (Santa Cruz, CA, USA). A polyclonal antibody against the phosphorylated form of Syk pTyr525/526 was purchased from Pierce (Rockford, IL, USA). An antibody against β-actin conjugated horseradish peroxidase (HRP) and rabbit IgG conjugated HRP were purchased from ZSGB-Bio (Beijing, China). The Syk inhibitor herbimycin A (88-H2030-35A) was purchased from Usbio (St. Louis, MO, USA). The NF-κB inhibitor Pyrrolidinedithiocarbamate Ammonium (PDTC) (93-1676-100) was purchased from Biovision (San Francisco,USA). The sodium salt of N-acetylneuraminic acid, Neuraminidase from *Clostridium perfringens* was purchased from Sigma-Aldrich (St. Louis, MO, USA). Protease inhibitor and phosphatase inhibitor cocktails, enhanced chemiluminescence (ECL) western blot detection reagents, and IFN-γ enzyme-linked immunosorbent assay (ELISA) kits were purchased from BOSTER (Wuhan, China).

### NK cell preparation and activation

Mice were sacrificed by cervical dislocation and their spleen were collected.Splenic leukocytes were obtained from mice using density iodixanol gradient centrifugation (800×g, 30min). NK cells were separated and purified from splenic leukocytes using a MACS separator, according to the manufacturer’s instructions. About 3×10^5^ purified NK cells were obtained from one mouse. The proportion of NK-enriched cells was >90% as determined by flow cytometry using the CD49(DX5)-FITC mAb.

NK cells were stimulated for 1 or 16h with NDV 7793 (25HU/10^5^cells), recombinant HN (500ng/mL), or IFN-γ (500ng/mL). Cells were collected by centrifugation (300–400×g, 10min, 4°C), washed twice in PBS, and used in western blotting or flow cytometry experiments. Supernatants were collected and IFN-γ concentrations determined by ELISA, according to the munufacturer’s protocol.

### Blocking experiments

NK cells were cultured in the presence of herbimycin A (250ng/mL), PDTC (500ng/mL), or neuraminidase (250ng/mL) at 28°C for 1h, followed by twice washings with PBS. Cells were then stimulated for 1 or 16h with NDV 7793 (25HU/10^5^cells), recombinant HN (500ng/mL), or IFN-γ (500ng/mL). The presence of these drugs had no effect on lactate dehydrogenase (LDH) levels, a stable cytosolic enzyme released upon cell lysis, in NK cells.

### Determination of IFN-γ concentrations

We used a specific ELISA kit to determine IFN-γ levels in harvested supernatants, according to the manufacturer’s recommendations. Results are presented as means from triplicate cultures.

### Western blotting analysis

To assess TRAIL expression levels, cell lysates were suspended in Tris-glycine sodium dodecyl sulfate (SDS) sample buffer. Western blotting was conducted using antibodies against TRAIL and β-actin (as an interal control). We used HRP-conjugated anti-mouse IgG as a secondary antibody. Protein bands were visualized by ECL.

To assess Syk and IκBα phosphorylation levels, splenic NK cells were incubated with HN for varying periods, collected, and washed three times with PBS. Cells were lysed with lysis buffer (50mM Tris-HCl pH8.0, 1% Nonidet P-40, 150mM NaCl, 5mM EDTA, protease inhibitor cocktail, and phosphatase inhibitor cocktail). Lysates were resuspended in Tris-glycine SDS sample buffer, and western blotting conducted with antibodies against Syk, pSyk, IκBα, and pIκBα. The VersaDoc imaging system (Bio-Rad) was used to quantify band intensities.

### Flow cytometry

Splenic NK cells from control or IFN-R1^-/-^ mice were stimulated with recombinant HN (500ng/mL) or NDV 7793 (25HU/10^5^cells) for 16h, and then incubated with a PE-conjugated antibody against TRAIL for 1h on ice. Cells were then washed with cold PBS prior to flow cytometry. For the NKp46 competition binding assay, cells were cultured in the presence or absence of recombinant HN (500ng/mL) for 1h, and then treated with a PE-conjugated antibody against NKp46. Cells were washed with cold PBS prior to flow cytometry.

### Statistical analysis

Each experiment was repeated three times. Data were analyzed using SPSS 16.0. We used analysis of variances (ANOVA), followed by Fisher’s least significant difference (LSD) test to compare the significance between different groups.

## Results

### The IFN-γ-independent TRAIL pathway exists in NDV-stimulated NK cells

**Fig 1:**
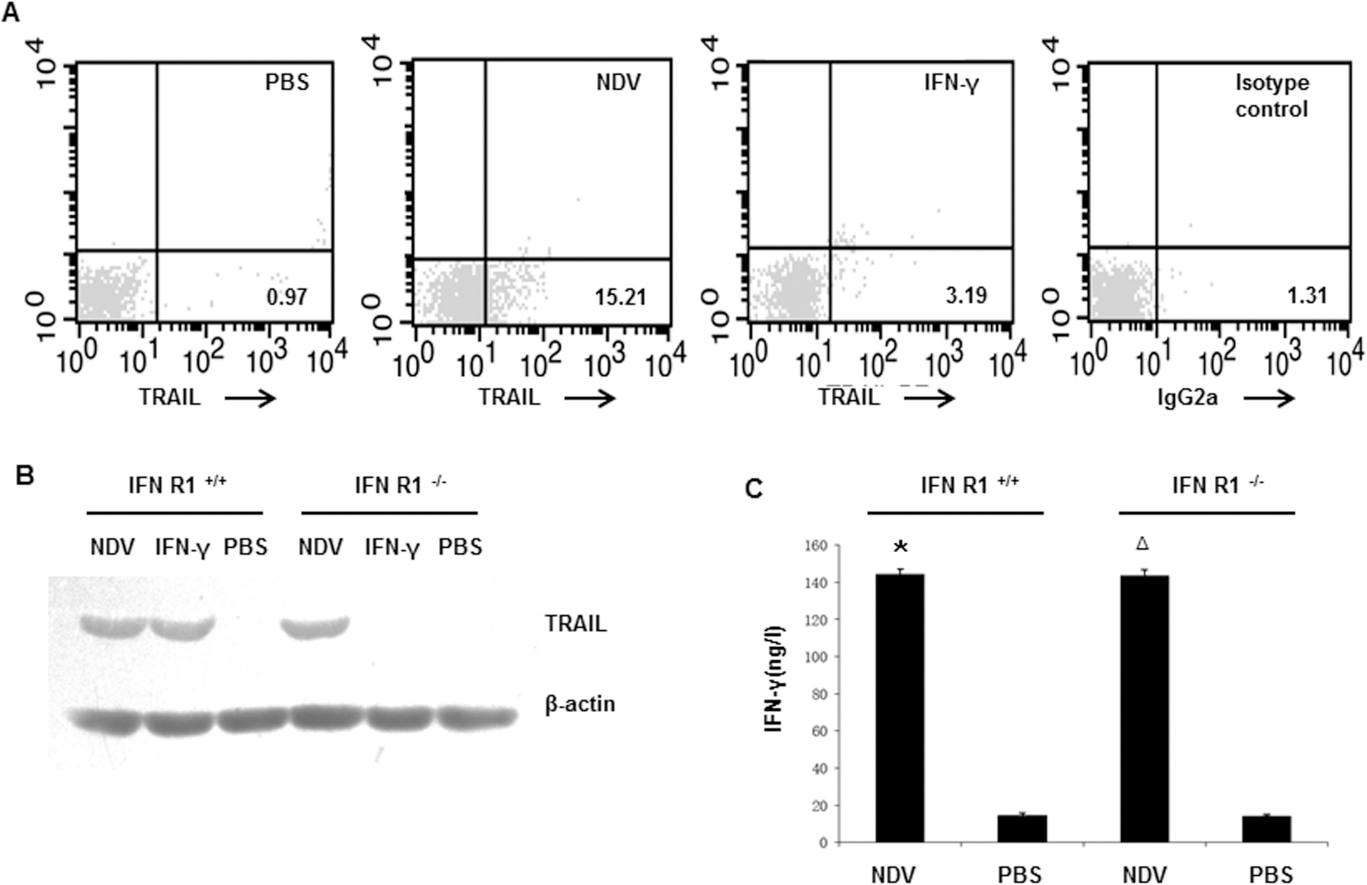
TRAIL expression in NDV-stimulated NK cells of mice where IFN-γ receptor one was deficient. (A) Purified splenic NK cells from IFN-R1^-/-^ mice were stimulated with NDV 7793 or IFN-γ. Cells were stained with a PE-conjugated monoclonal antibody (mAb) against TRAIL and then examined by flow cytometry. PBS-treatment NK cells were used as negative controls. Results are representative of three independent experiments. (B) Purified splenic NK cells from IFN-R1^+/+^ and IFN-R1^-/-^ mice were stimulated with NDV 7793 or IFN-γ. Cellular proteins were extracted and subjected to western blotting using a mAb against TRAIL. Results are representative of three independent experiments. (C) The concentration of IFN-γ in supernatants was determined by ELISA. Values represent the mean + standard deviation from triplicate wells, with results obtained from three independent experiments. *P<0.01 and ^Δ^*P*<0.01 *vs*. unstimulated NK cells in IFN-R1^+/+^ and IFN-R1^-/-^ mice, respectively.

Exposure of NK cells to NDV induces antitumor effects that is partly mediated by the upregulation of TRAIL effectors, via an IFN-γ-dependent pathway [18].To evaluate the upregulated expression of TRAIL exists without the participation of IFN-γ, we investigated whether stimulation with NDV resulted in an increase in TRAIL^+^ NK cells in IFN-R1^-/-^ mice, and compared TRAIL expression levels between IFN-R1^+/+^ and IFN-R1^-/-^ mice. In IFN-R1^-/-^ mice, a marked and rapid increase in the number of TRAIL^+^ NK cells was observed after treatment by NDV in comparison with those by PBS or IFN-γ treatment (Fig 1A). After stimulation with NDV for 16h, TRAIL expression was induced in IFN-R1^+/+^ and IFN-R1^-/-^ NK cells (Fig 1B). As we expected, TRAIL expression was induced in IFN-R1^+/+^ NK cells by IFN-γ identified before[18]; however, IFN-γ had no effect on TRAIL induction in IFN-R1^-/-^ NK cells(Fig 1B). NDV directly stimulated NK cells, resulting in upregulated TRAIL expression when the IFN-γ signaling pathway was absent. We observed no differences in the levels of IFN-γ secreted by IFN-R1^-/-^ and IFN-R1^+/+^ NK cells following stimulation with NDV (Fig 1C). It was indicated the absence of the IFN-γ receptor one does not influence or inhibit signal regulation events associated with the secretion and production of IFN-γ.

### NDV HN directly triggers TRAIL production in IFN-R1^-/-^ NK cells

**Fig 2:**
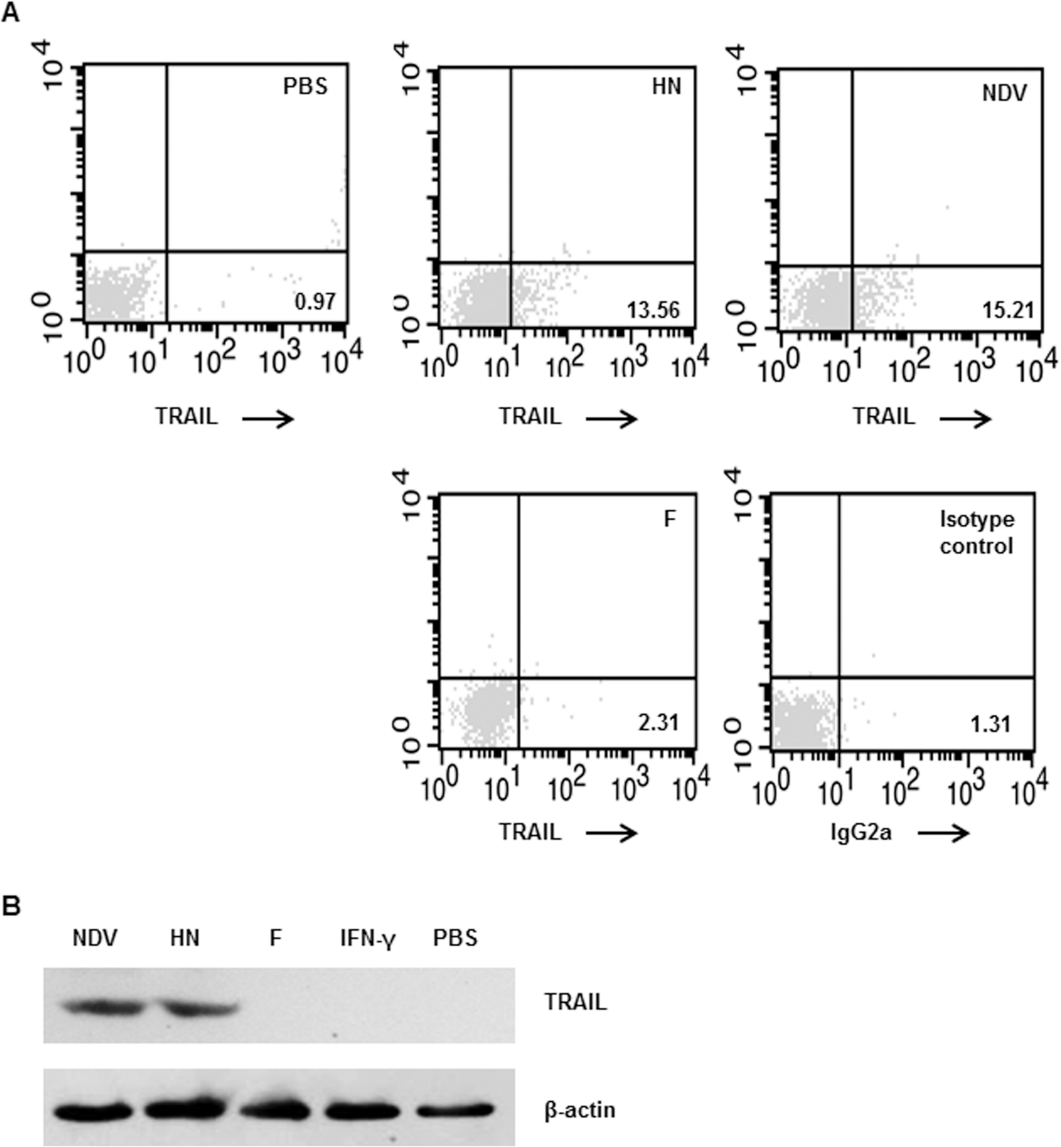
TRAIL expression in IFN-R1^-/-^ NK cells following stimulation with NDV-HN. (A) Purified murine splenic IFN-R1^-/-^ NK cells were incubated with NDV 7793, recombinant HN protein, or F protein, respectively. Cells were stained with PE-conjugated anti-TRAIL mAbs. Unstimulated NK cells were used as negative controls. (B) Total cellular protein was extracted and subjected to western blotting using a mAb against TRAIL; β-actin was used as an internal loading control. Results are representative of three independent experiments.

To determine which component of the virus particles is responsible for the IFN-γ-independent TRAIL pathway, we used NDV recombinant HN and F protein, respectively, to stimulate NK cells. We observed increased numbers of TRAIL^+^ NK cells in IFN-R1^-/-^ mice when NDV or HN were used as stimuli. However, there was no change on the numbers of TRAIL^+^ NK cells in IFN-R1^-/-^ mice when F protein was used stimuli (Fig 2A). Western blotting analysis confirmed these findings (Fig 2B).

### HN-induced TRAIL upregulation occurs via direct binding to NKp46

**Fig 3:**
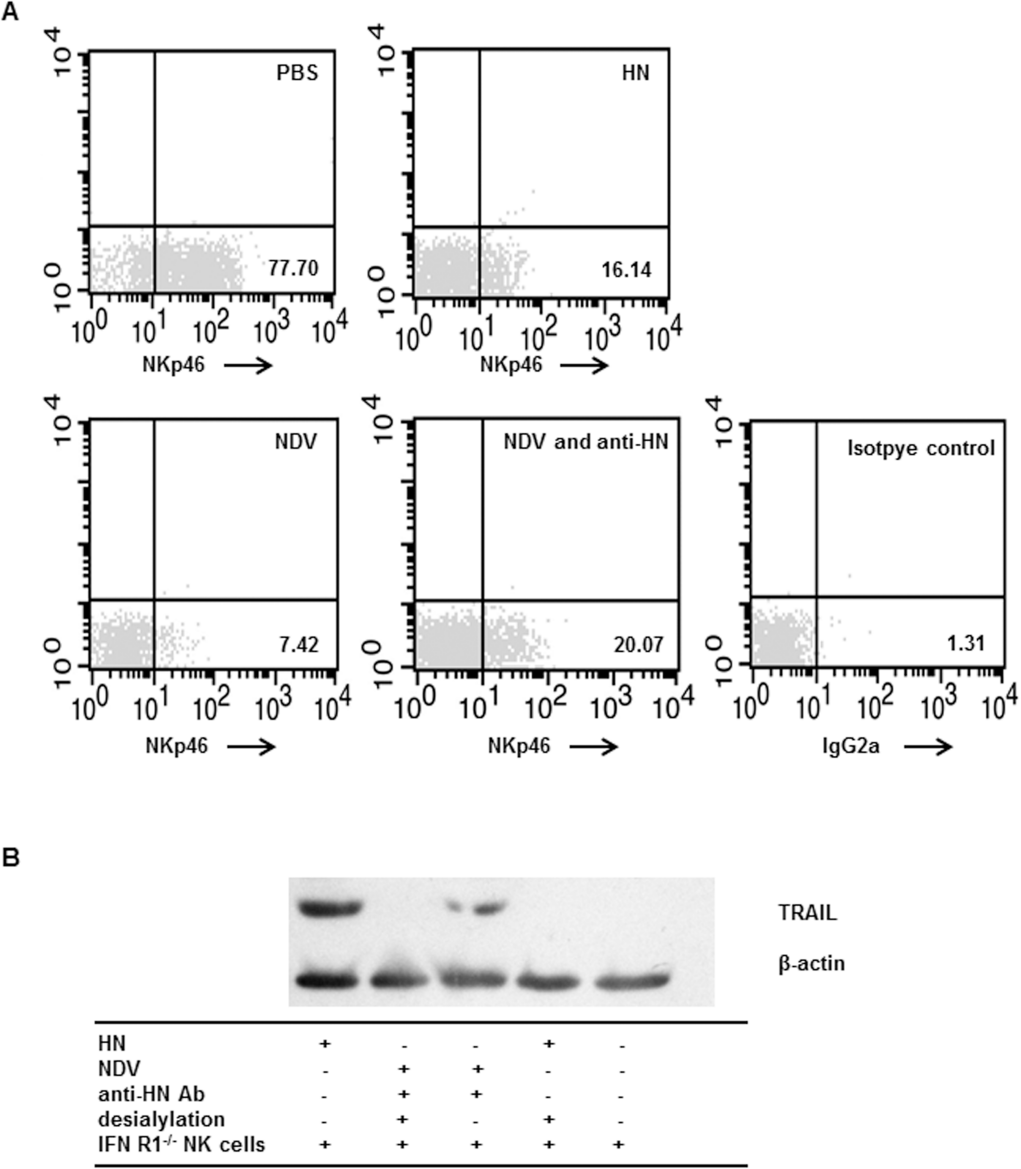
Recombinant NDV HN upregulated TRAIL expression in NK cells by binding to NKp46. (A) Purified murine splenic IFN-R1^-/-^ NK cells were exposed to HN, NDV 7793, or a mixture of NDV 7793 and neutralizing antibodies against HN, and then incubated with a PE-conjugated mAb against NKp46. PBS-treatment NK cells were used as negative controls. (B) TRAIL protein expression in NK cells where NKp46 was recognized by NDV HN protein. Purified IFN-R1^-/-^ NK cells were treated with neuraminidase for desialylation prior to stimulation with HN. Alternatively, IFN-R1^-/-^ NK cells were treated with a mixture of NDV 7793 and neutralizing antibodies against HN. Cellular proteins were extracted and subjected to western blotting using a mAb against TRAIL. Results are representative of three independent experiments.

HN of NDV serves as a ligand structure for NKp46 of human NK cells[25]. To determine whether direct interactions between HN and NKp46 mediated TRAIL upregulation in NK cells, we conducted flow cytometry assays in conjunction with NKp46 competition binding assays. The binding ability of the NKp46 mAb to NK cells was abrogated when NK cells were pre-incubated with NDV or recombinant HN. When NK cells were pre-incubated with a mixture of NDV and neutralizing antibodies against HN, the binding ability of NKp46 mAb was partially abrogated, thereby indicating interactions between HN and NKp46 (Fig 3A). To investigate whether the recognition of NKp46 by HN can activate NK cells and upregulate TRAIL expression, the blockade of binding between HN and NKp46 in the present of anti-HN antibody and by desialylation treatment on NK cells were used. We observed that upregulated TRAIL expression was then efficiently blocked (Fig 3B). However, the portion of the HN protein that participates in the molecular interaction between NDV and NK cells remains unknown.

### The correlation between TRAIL upregulation and Syk, NF-κB pathways in IFN-R1^-/-^ NK cells

**Fig 4:**
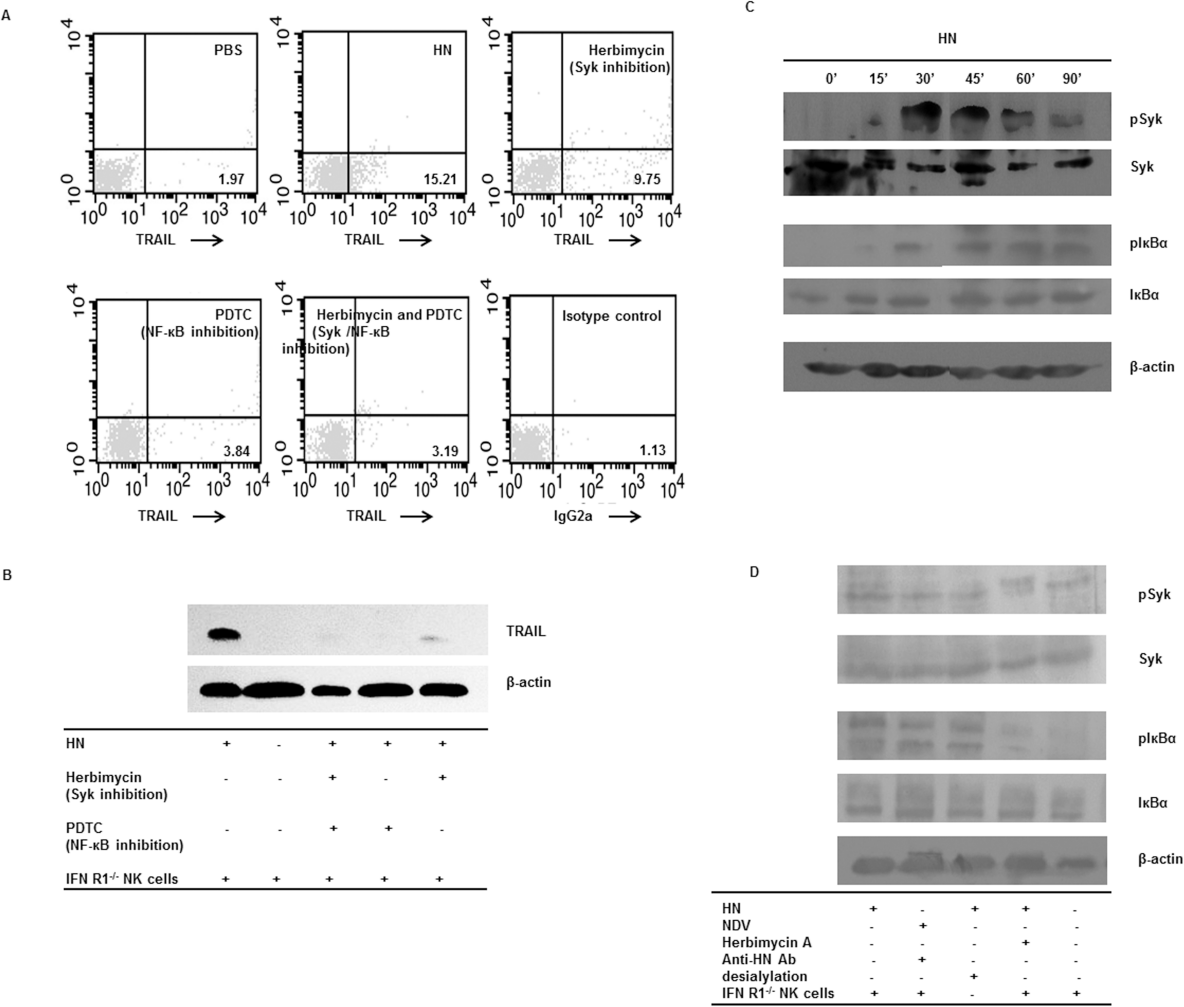
The correlation between TRAIL upregulation and Syk, NF-κB pathways in IFN-R1^-/-^ NK cells. (A) HN-induced TRAIL expression in NK cells was mediated by spleen tryosine kinases (Syk) and NF-κB pathways. Purified murine splenic IFN-R1^-/-^ NK cells were pre-cultured with herbimycin A or PDTC, and then incubated with soluble recombinant NDV HN. Cells were stained with a PE-conjugated mAb against TRAIL. PBS-treatment NK cells were used as negative controls. (B) Cellular proteins were extracted and subjected to western blotting with a mAb against TRAIL. Results are representative of three independent experiments. (C) Phosphorylation of Syk and IκBα in HN-induced IFN-R1^-/-^ NK cells. Purified IFN-R1^-/-^ NK cells were incubated with recombinant NDV HN for 15, 30, 45, 60, and 90min. Cellular proteins were extracted, and Syk, phosphorylated Syk (pSyk), IκBα, and phosphorylated IκBα (pIκBα) were detected by western blotting. β-actin was used as a loading control. (D) Purified IFN-R1^-/-^ NK cells were pre-cultured with the sodium salt of N-acetylneuraminic acid, 2,3-dehydro-2-deoxy, or herbimycin A, prior to incubation with HN or a mixture NDV 7793 and neutralizing antibodies against HN. Cellular proteins were extracted, and Syk, pSyk, IκBα, and pIκBα were detected by western blotting. β-actin was used as a loading control. Results are representative of three independent experiments using different mice.

To investigate whether HN-induced upregulation of TRAIL was dependent on Syk and NF-κB activity, IFN-R1^-/-^ NK cells were pre-incubated with herbimycin A used as specific Syk kinase inhibitors and PDTC used as NF-κB inhibitors following stimulation with HN. These two inhibitors significantly blocked HN-induced TRAIL expression (Fig 4A and 4B). Our results suggest that TRAIL expression in HN-stimulated IFN-R1^-/-^ NK cells was downregulated when Syk or NF-κB activity was inhibited.

To determine whether HN stimulation resulted in increased activation of Syk in IFN-R1^-/-^ NK cells, we assessed Syk and phosphorylated Syk (pSyk) levels. We observed an increase in pSyk levels 30 min after treatment with HN, peaking after 60 min, and then decreasing. There were no differences in Syk levels of IFN-R1^-/-^ NK cells at various time points after stimulation with HN (Fig 4C). To confirm Syk activation by KARs, NDV was treated with a neutralizing mAb against HN, while NK cells were treated with neuraminidase for desialylation. We found that levels of pSyk were clearly reduced following these treatments, compared with those in controls (Fig 4D). These results suggest that Syk plays an important role in NK cells stimulated with NDV and HN.

To investigate if the NF-κB pathway was involved in mediating HN stimulation of IFN-R1^-/-^ NK cells, we assessed levels of IκBα and phosphorylated IκBα (pIκBα) in the cytoplasm of NK cells. We found that phosphorylation of IκBα began to increase 30 min after treatment with HN, peaking after 60min, and then slowly declining after 90 min (Fig 4C). To identify if phosphorylation of IκBα occurred through the activation of KARs and Syk, we conducted blocking experiments. A clear decrease in phosphorylation levels of IκBα were observed for HN-stimulated NK cells pretreated with the various inhibitors compared with those in controls (Fig 4D). We showed that HN-induced expression of TRAIL in IFN-R1^-/-^ NK cells was dependent on the NF-κB signaling pathway.

## Discussion

TRAIL, also known as Apo2L, is a critical effector of immune cells for tumoricidal activity. TRAIL-mediated apoptosis is triggered *via* the recognition of the proapoptotic membrane death receptors DR4/DR5, which are only expressed on tumor cells, and is regulated by a variety of physiological and pharmacological inducers [27–33].

NDV is able to stimulate immunocyte-mediated antitumor effects, through TRAIL expression, *in vivo* and *in vitr*o. TRAIL induction has been observed on the surface of various NDV-stimulated lymphocytes, such as macrophages, PBMC and NK cells [21, 25, 34]. In macrophages, TRAIL is a major effector and mediator of cell-to-cell contact in the killing of tumor cells following stimulation with NDV [17]. However, the mechanism(s) by which NDV induces TRAIL expression have not been elucidated.

It was previously shown that NDV is a strong inducer of type I and II IFNs in mouse and human lymphocytes [16, 35]. During NDV stimulation, large quantities of type I and II IFNs are released to induce TRAIL-dependent apoptosis [36–38]. Several IFN signaling factors, including signal transducer and activator of transcription 1 (STAT1), interferon regulatory factor 1 (IRF1) and interferon regulatory factor 3 (IRF3), were found to mediate the effects of IFNs on the TRAIL promoter and induce apoptosis [39–42]. However, in the current study, we saw that TRAIL expression was upregulated in NDV-induced NK cells from IFN-R1^-/-^ mice. These results reflected those we reported previously, where TRAIL expression was not completely inhibited by neutralizing antibodies against IFN-γ[18]. Our findings suggest the existence of an IFN-γ-independent TRAIL induction pathway. A similar phenomenon was seen in mammalian I IFN receptor-deficient cells, with apoptosis induced by a mammalian reovirus[43]. These results indicated that the apoptotic response triggered by TRAIL might be mediated by IFN independent way in response to viral particles.

The HN envelope protein of NDV is able to mediate the binding of the viral particle to host cells. It was found that HN acts as a ligand for NKp46 and NKp44 receptors on human NK cells, directly activating NK cells and contributing to the antitumor effects of NDV [25]. In the current study, we showed that a recombinant HN protein of NDV was able to trigger upregulated expression of TRAIL in NK cells. This direct activation occurred without the participation of IFN-γ in IFN-R1^-/-^ NK cells. We also found that an existing form of HN, which was expressed on the surface of NDV-infected tumor cells, was able to induce upregulation of TRAIL expression. HN-dependent TRAIL expression was in addition to that induced by tumor cells, which possess the ability to induce TRAIL-mediated apoptosis (data not shown). It is unclear whether the binding of HN to NKp46 activates the production of other cytokines which TRAIL induction pathway depends on. Based on the conclusion that the direct activation of NK cells by NDV results in increased cytotoxicity against tumor cells *in vivo* [25], our results suggest that TRAIL might play a role in contributing to these increased cytotoxicity.

The HN protein mediates viral absorption by recognizing sialic acid-containing molecules on the surface of host cells [44]. The viral recognition site comprises terminal sialic acid residues attached to galactose [45]. Desialylation of NKp44 and NKp46 on human NK cells eliminates the ability of these cells to interact with HN. The neuraminidase inhibitor, Neu5Ac, which acts upon the active site of HN, abrogates NDV-mediated oncolytic activity and eliminates the production of functional TNF-α and IFN-γ [25]. We showed that desialylation of NKp46 on IFN-R1^-/-^ NK cells inhibited increased TRAIL production. This finding was consistent with previous results, where HN served as the ligand of NKp44 and NKp46 [17]. However, the site(s) in HN that recognize the sialic acid residues of NKp44 and NKp46 remain to be elucidated. The functions of sialic acid and neuraminidase in the interaction of HN and NKp44 and/or NKp46 require further investigation.

The upregulation of TRAIL following NDV stimulation was mediated by the binding of HN to the NKp46 receptor of mice NK cells. NKp46 integrated with killer inhibition receptors (KIRs) to regulate the cytotoxicity of NK cells against transformed cells and virus-infected cells. In addition, NKp46 and KIR interactions modulate the release of cytokines critical to the immune response. Enhanced expression of NKp46, accompanied by augmented TRAIL expression, was observed in NK cells from patients chronically infected with hepatitis B virus [46], and in T cells pretreated with phorbol 12-myristate 13-acetate and ionomycin [47]. NKp46 is critical to the activation of NK cells, in response to NDV [25]. NKp46 which lacks an activating cytoplasmic component can transmit a cell activating signal via CD3 ζ chain, thereby affecting final cytotoxicity [48, 49]. CD3ζ chain can be activated by NDV, as shown in lacZ-inducible reporter cells [25]. Little is known about the downstream intracellular signaling cascades involving the CD3 ζ chain that trigger NK cell cytotoxicity *via* TRAIL. We saw that HN-treated NK cells contained significantly higher levels of phosphorylated Syk (pSyk) and phosphorylated IκBα (pIκBα), which resulted in increased TRAIL production. Blocking the binding of HN to NKp46 resulted in reduced pSyk and pIκBα levels. Exposure to herbimycin A, a known Syk inhibitor, or to PDTC, an NF-κB inhibitor, partially inhibited the production of TRAIL. However, in our system, to avoid any stimulation that may cause TRAIL upregulation, the freshly isolated NK cells were obtained by purification of negative selection and then were cultured in RPMI-1640 media with the absent of sera. NK cells obtained can only keep activated within 30 hours in these environments. Therefore, siRNA or shRNA experiments and LacZ inducible detection were limited to perform in our system for further evidences about intercellular signal cascades. The findings in our work suggest that activation of Syk were required signaling events in the regulation of TRAIL in HN-stimulated NK cells. However, conflicting results have been observed with influenza viruses. The hemagglutinin (HA) protein of influenza viruses inhibits the cytotoxicity of human NK cells. This is mediated by NKp46, with downregulated expression of the CD3 ζ chain, and reduced levels of pSyk and pERK [50]. These conflicting findings could explain why innate immune defense directed by NK cells likely contribute to the pathogenesis of influenza virus, but limit the spread of NDV. Syk kinases are thought to dock with ITAM-containing molecules that are involved in perforin- and granzyme-associated cytotoxicity [51, 52]. Our results indicate that Syk kinases are involved in a specific signal cascade mediated by TRAIL production. Therefore, we postulate that there are two alternative routes of NK cell-mediated cytotoxicity that share signaling elements.

In summary, we have provided evidence that NDV HN directly triggers the upregulation of TRAIL in murine NK cells *via* an IFN-γ-independent pathway. Both Syk and NF-κB pathways play a role in the regulation of TRAIL expression in HN-stimulated NK cells. These findings indicate that the interaction between HN and NKp46 induces the upregulation of TRAIL *via* the modulation of Syk and NF-κB activity. Our results highlight the immunostimulatory capabilities of NDV upon oncolytic activity.
